# Advantages of using a polymeric clip versus an endoloop during laparoscopic appendectomy in uncomplicated appendicitis: a randomized controlled study

**DOI:** 10.1186/s13017-023-00507-6

**Published:** 2023-06-29

**Authors:** Kil-yong Lee, Jaeim Lee, Youn Young Park, Seong Taek Oh

**Affiliations:** 1 Division of Coloproctology, Department of Surgery, Uijeongbu St. Mary’s Hospital, College of Medicine, The Catholic University of Korea, 271, Cheonbo-ro, Uijeongbu-si, Gyeonggi-do 11765 Republic of Korea; 2 Department of Surgery, Kyung Hee University Hospital at Gangdong, Kyung Hee University School of Medicine, Seoul, Republic of Korea

**Keywords:** Appendicitis, Appendectomy, Laparoscope, Surgical clip

## Abstract

**Background:**

Polymeric clips are easy to apply, but whether they present more advantages than endoloops is unclear. This single-center, open-label, randomized controlled trial study was conducted to compare the advantages of using a polymeric clip versus an endoloop in terms of the surgical time.

**Methods:**

Adult patients who were diagnosed with acute appendicitis without perforation on preoperative abdominal computed tomography and underwent laparoscopic appendectomy between August 6, 2019, and December 26, 2022, were included. Single-blinded randomization was performed in a 1:1 ratio between the endoloop and polymeric clip groups. The primary endpoint was the difference in surgery time between the polymeric clip and endoloop groups. The secondary endpoints were the difference in the application time of each instrument, difference in operation and anesthesia fees, as well as the frequency of complications.

**Results:**

The completed trial included 104 and 103 patients in the polymeric clip and endoloop groups, respectively. The median surgery time with a polymeric clip was shorter than that with an endoloop; however, the difference was not significant (18 min 56 s vs 19 min 49 s, *p* = 0.426). Interestingly, the median time from applying the instrument to appendiceal cutting in the polymeric clip group was significantly shorter than that in the endoloop group (49.0 s vs 84.5 s, *p* < 0.001). No significant difference was observed between the two groups in terms of surgical (*p* = 0.120) and anesthetic (*p* = 0.719) costs, as well as the total number of postoperative complications (*p* > 0.999).

**Conclusion:**

A polymeric clip is a safe instrument that can reduce the time from applying the instrument to appendiceal cutting, although it does not affect the overall surgical time and operation fee when performing laparoscopic appendectomy for uncomplicated appendicitis.

*Trial registration*: KCT0004154.

## Background

When performing a laparoscopic appendectomy, an endoloop, an endostapler, or a polymeric clip can be used to ligate and resect the appendiceal stump [1, 2]. Of these, the endostapler makes resection of the appendiceal stump easier than the other instruments, but has the disadvantage of being expensive [3]. The cost has further implications for patients in Korea because acute appendicitis is included in Diagnosis-Related Group Payment (DRG) and the expense is reimbursed according to the National Health System tariffs based on the surgery name (e.g., appendectomy) regardless of the device. Therefore, there are restrictions on the use of endostaplers during laparoscopic appendectomy.

During the process of ligating the appendiceal base, an endoloop is relatively difficult to handle, whereas a polymeric clip has the advantage of being easy to apply [4]. The safety of using polymeric clips during laparoscopic appendectomy is well known [5]. Most studies have reported that using polymeric clips can shorten the surgical time compared with that using endoloops [6–8]. Recent guidelines also state that using polymeric clips may be the cheapest and easiest method with shorter operative times [9]. Furthermore, in the study by Al-Termini et al., using polymeric clips showed fewer overall complications than using endoloops [10]. However, clear evidence of the advantages of polymeric clips over endoloops is lacking owing to a dearth of randomized controlled trial (RCT) studies on the above-mentioned advantages [11].

Therefore, we conducted an RCT study to compare the operative times of laparoscopic appendectomy for uncomplicated appendicitis using polymeric clips to those using endoloops.

## Methods

This was a single-center, open-label, RCT. This study was approved by the Institutional Review Board (IRB) of the Catholic University of Korea and performed in accordance with the guidelines and regulations of the IRB. Informed consent was obtained from all the included patients. The study was conducted after registration with Korea Clinical Research Information Service (KCT0004154).

### Patients

Nonpregnant adult patients aged 19–70 years who were diagnosed with acute appendicitis without perforation on preoperative abdominal computed tomography (CT) were eligible. Consecutive patients who underwent surgery between August 6, 2019, and December 26, 2022, in Uijeongbu St. Mary’s Hospital were included, and the follow-up date of the last patient was January 04, 2023. The exclusion criteria were as follows: (1) history of abdominal surgery; (2) open conversion; (3) surgery other than laparoscopic appendectomy; (4) suspicion of perforation or periappendiceal abscess in the surgical field; (5) difficulty in applying a polymeric clip owing to a thick appendiceal base or severe inflammation; and (6) the final biopsy result not being appendicitis.

### Sample size calculation

The sample size calculation was based on the results of a previous study that compared the use of polymeric clips with that of endoloops [12]. In this study, the operative time using polymeric clips and endoloops was 59 min and 68 min, respectively. Therefore, considering that a clinically meaningful difference in the average surgical time between using polymeric clips and endoloops is 9 min, the standard deviation calculated through the range of surgical time was confirmed to be 22.5 min. A sample size of 98 in each group achieved 80% power to reject the null hypothesis of equal means when the population mean difference was 9 min with a standard deviation for both groups of 22.5 min and a significance level (alpha) of 0.050 using a two-sided two-sample equal-variance t-test. Considering a dropout rate of 5%, 208 patients (104 patients per group) were included in this study.

### Randomization and blinding

Using SAS ver 9.4 (SAS Institute Inc., NC, Cary, USA) for Microsoft Windows, random assignment numbers were generated, and a random assignment envelope was created. Before a polymeric clip or an endoloop was applied, the envelope was opened, and the patients were assigned to either the intervention (polymeric clip) or the control (endoloop) group. This was a single-blinded study where the use of polymeric clips or endoloops was not blinded to the investigators, including outcome accessors, but to the patients.

### Primary and secondary endpoints

The primary endpoint was the difference in surgical time between the polymeric clip and endoloop groups. The secondary endpoints were differences in the time from the introduction of each instrument to appendiceal base cutting, operation and anesthesia fees, as well as the frequency of complications.

### Procedure

The amount of time from the initiation of skin incision to the completion of skin suturing was defined as the total surgical time. To minimize bias in surgical timepoints, the surgical time was subdivided into skin incision, mesoappendiceal dissection (from start to completion time), polymeric clip or endoloop application (from start to appendix cutting time), and skin suture completion time.

In both groups, surgery was performed through three abdominal incisions (one with a 12-mm trocar and two with 5-mm trocars) and using a 5-mm scope. The mesoappendix was dissected using an energy and/or monopolar device. When ligating the appendiceal base, two polymeric clips (Gems-clip Plus, size: XL [12 mm]) and one endoloop (Gemsloop-PGLA) were used in the intervention and control groups, respectively. After the appendix was cut, a drainage tube was placed according to the surgeon's judgment. The specimen was extracted using a plastic bag via the 12-mm trocar site and the trocar was then removed, followed by suturing the skin. The operation and anesthesia fees for each surgery were then calculated.

Postoperative acute complications were monitored during hospitalization. If the patients agreed and there were no specific complications, they were discharged on the second postoperative day.

One to two weeks after surgery, the patients were checked for postoperative complications during an outpatient visit.

### Statistical analysis

Continuous variables are presented as mean and standard deviation in cases of normal distribution, as well as median and interquartile range (IQR) values in cases of non-normal distribution. Categorical variables are presented as frequencies and percentages. To evaluate the difference between the two groups in continuous variables, the normality test was performed followed by the independent T-test or Wilcoxon's rank sum test. For categorical data, a chi-square test or Fisher's exact test was performed. All statistical analyses were performed using SPSS version 21.0 for Windows (IBM Corp, Armonk, NY, USA), and the significance level was set to 0.05.

## Results

**Fig. 1:**
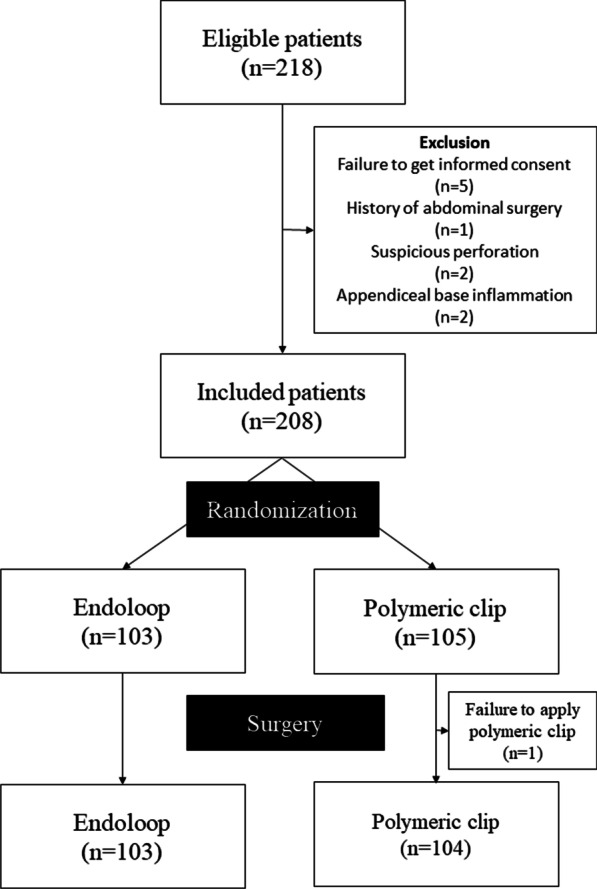
Flowchart of the patient selection

From August 6, 2019, to December 26, 2022, of the 218 eligible patients, 10 were excluded and 208 were included in this study. After randomization, 103 and 105 patients were assigned to the endoloop and polymeric clip groups, respectively. However, polymeric clip failure was observed in one patient. Thus, only 104 patients were included in the polymeric clip group (Fig. 1). For the patient who had polymeric clip failure, appendectomy was performed using an endoloop, but this patient was not included in the analysis.

No differences were observed between the two groups in terms of age (*p* = 0.513), sex (*p* = 0.519), or body mass index (BMI) (*p* = 0.630). Preoperative laboratory findings also showed no significant difference between the two groups in terms of the white blood cell count (*p* = 0.783) and C-reactive protein level (*p* = 0.656) (Table 1).

**Table 1:** Baseline characteristics of the patients in the two groups

	Total	Endoloop (n = 103)	Polymeric clip (n = 104)	p-value
Age (years)	37 (IQR 26–49)	35 (IQR 26–49)	37 (IQR 27.3–49.8)	0.513 (W)
Sex				0.519 (C)
Male	120 (58.0%)	62 (60.2%)	58 (55.8%)	
Female	87 (42.0%)	41 (39.8%)	46 (44.2%)	
Height (cm)	169.5 (IQR 162.0–174.2)	170.1 (IQR 162.6–175.0)	168.5 (IQR 162.0–174.0)	0.360 (W)
Weight (kg)	69.3 (IQR 58.8–77.0)	70 (IQR 58–80)	67.9 (IQR 60–75)	0.432 (W)
Body mass index (kg/m^2^)	24.1 (IQR 22.0–26.6)	24.1 (IQR 21.6–27.1)	24.1 (IQR 22.0–26.2)	0.630 (W)
Systolic blood pressure (mmHg)	134.6 ± 19.2	132.9 ± 19.0	136.4 ± 19.4	0.200 (T)
Diastolic blood pressure (mmHg)	82 (IQR 73–92)	83 (IQR 73–93)	81 (IQR 74–91)	0.928 (W)
Body temperature (℃)	36.8 (IQR 36.5–37.3)	36.9 (IQR 36.5–37.4)	36.8 (IQR 36.4–37.3)	0.271 (W)
Pulse rate (/min)	88.3 ± 17.4	88.4 ± 17.9	88.2 ± 16.9	0.936 (T)
Hypertension	20 (9.7%)	8 (7.8%)	12 (11.5%)	0.358 (C)
Diabetes	6 (2.9%)	3 (2.9%)	3 (2.9%)	> 0.999 (F)
White blood cell (× 10^3^/uL)	13.2 ± 4.2	13.2 ± 4.0	13.1 ± 4.4	0.783 (T)
Segment neutrophil count (%)	79.4 (IQR 71.4–84.3)	79.2 (IQR 71.4–84.0)	80.2 (IQR 71.8–84.9)	0.448 (W)
C-reactive protein (mg/dl)	0.8 (IQR 0.2–3.0)	0.8 (IQR 0.2–3.8)	0.8 (IQR 0.2–2.2)	0.656 (W)
Total protein (g/dl)	7.3 (IQR 7.0–7.6)	7.3 (IQR 7.0–7.7)	7.3 (IQR 7.0–7.6)	0.306 (W)
Albumin (g/dl)	4.7 (IQR 4.5–5.0)	4.7 (IQR 4.5–5.0)	4.7 (IQR 4.5–5.0)	0.474 (W)

IQR, interquartile range

*p*-value: Independent *t*-test (T) or Wilcoxon rank sum test (W)

*p*-value: Chi-square test (C) or Fishers exact test (F)

As the primary endpoint, the median surgery time of all patients was 19 min 25 s (IQR 15 min 46 s–25 min 30 s). The median surgery time of the polymeric clip group was 18 min 56 s (IQR 15 min 19 s–24 min 01 s), which was shorter than 19 min 49 s (IQR 16 min 08 s–25 min 23 s) in the endoloop group. However, no significant difference was observed (*p* = 0.426) (Table 2).

**Table 2:** Operation-related outcomes

	Total	Endoloop (n = 103)	Polymeric clip (n = 104)	p-value
Operative time (mm:ss)	19:25 (IQR 15:46–25:30)	19:49 (IQR 16:08–25:23)	18:56 (IQR 15:19–24:01)	0.426 (W)
Time to mesoappendix dissection (seconds)	114 (IQR 60–178.5)	114.5 (IQR 59.0–170.5)	111.0 (IQR 61.0–185.0)	0.767 (W)
Time from applying the instrument to appendiceal cutting (seconds)	68 (IQR 48–93)	84.5 (IQR 68.8–121.5)	49.0 (IQR 36.0– 66.0)	< 0.001 (W)
Anesthetic time (minutes)	40 (IQR 35–50)	40.5 (IQR 35.0–50.0)	40.0 (IQR 35.0–50.0)	0.886 (W)
Surgical cost (₩)	741,585 (IQR 729,925–755,825)	738,325 (IQR 725,835–756,413)	744,938 (IQR 732,760–755,815)	0.12 (W)
Anesthetic cost (₩)	215,527 (190,288–265,161)	227,911 (190,288–265,161)	203,988 (IQR 188,788–265,686)	0.719 (W)
Intraperitoneal drainage (yes)	67 (32.4%)	29 (28.2%)	38 (36.5%)	0.197 (C)
Hospital stay (days)	2	2	2	0.676 (W)
Total postoperative complications	7 (0.5%)	3 (2.9%)	4 (3.8%)	> 0.999 (F)
Postoperative ileus	1 (0.5%)	0	1 (1.0%)	> 0.999 (F)
Wound complication (superficial surgical infection)	6 (2.9%)	3 (2.9%)	3 (2.9%)	> 0.999 (F)

IQR, Interquartile range

*p*-value: Wilcoxon rank sum test (W)

*p*-value: Chi-square test (C) or Fishers exact test (F)

Interestingly, the median time from applying the instrument to appendiceal cutting was significantly shorter in the polymeric clip group (polymeric clip vs. endoloop: 49 s vs. 84.5 s, *p* < 0.001) (Table 2).

No difference in terms of time to mesoappendiceal dissection (*p* = 0.767) and total anesthesia time (*p* = 0.886) was observed between the two groups. Moreover, no significant difference was observed between the two groups in terms of surgical (*p* = 0.120) and anesthetic (*p* = 0.719) costs (Table 2).

Seven postoperative complications occurred in both groups. Postoperative ileus occurred in one patient in the polymeric clip group. Wound complications occurred in six patients (2.9%), with three patients in each group and no significant difference (*p* > 0.999) (Table 2).

## Discussion

In this study, we were unable to verify the superiority of the polymeric clip over the endoloop in laparoscopic appendectomy for uncomplicated appendicitis in terms of shortening the operative time. However, it was confirmed that the application time of the polymeric clip was shorter than that of the endoloop, and no difference in postoperative complications was observed.

Several studies have compared the use of polymeric clips, endoloops, and endostaplers for appendiceal base ligation [1, 11, 13]. Among these instruments, the endostapler was excluded from our study because of the limitations of DRG in performing laparoscopic appendectomy. According to a systematic review conducted by Makaram et al. comparing the use of endoclips and endoloops [13], the average operative time using endoloops and endoclips was 54.8 min (range 47–66 min) and 47.7 min (range 31.1–66 min), respectively, indicating that the use of an endoclip offers a shorter operative time. However, only three RCTs were included in this systematic review. In contrast, in a meta-analysis conducted by Knight et al. in 2019 [11], the operative time of the polymeric clip and endoloop groups was 37 min (range 26–65 min) and 39 min (range 44–76 min), respectively, without any significant difference between the two groups (*p* = 0.365). We conducted the study by focusing on the application time of the instrument. Although no difference in the overall operation time was observed, a significant difference in the application time was confirmed. It is noteworthy that a larger sample size was used in our study than in previous RCTs.

The advantage of using polymeric clips is that they can be applied precisely to the desired area. The endoloop needs to be tightened for ligation, with the disadvantage that the surrounding tissue may be pinched during the tightening process. In our study, the pre-knotted area was tightened in two cases, and the surrounding tissues (sigmoid mesocolon and terminal ileal tissue) were pinched together. Although it did not cause complications, it made intra-abdominal handling challenging, with disadvantages for accurate targeting.

One of the reasons for the failure to prove the superiority of the polymeric clip in reducing surgical time is that the overall time of laparoscopic appendectomy in our study was shorter than that in the study used as a reference for sample size calculation [12]. Although no significant difference between the two groups was observed, the overall time with the use of the polymeric clip seemed shorter than that with the use of the endoloop, which may prove that the surgical time with the use of the polymeric clip decreased when the study was conducted with a larger number of patients. Most importantly, when the application time was analyzed separately, although there was a minor difference of seconds, the application time of the polymeric clip was significantly shorter than that of the endoloop, proving the superiority of the polymeric clip.

The major limitation of the polymeric clip is that it can be difficult to apply to a thick appendiceal base or an appendiceal base with severe and friable inflammation [1, 14]. In our study, one case in the polymeric clip group was excluded because the clip was difficult to apply owing to appendiceal base thickening without inflammation. In the preoperative CT of the appendiceal base, the thickness of the base was 13 mm. As the maximum size of the polymeric clip is 16 mm, it is reasonable to select and use it in cases where the base thickness is less than 12 mm on preoperative CT [14].

A limitation of our study is that it was not double-blinded because the researcher included in the clinical trial performed the surgery. However, we attempted to avoid bias as much as possible by having a person unrelated to the study check the time difference and divide the time required for surgery for each procedure. Second, the study lacked empirical evidence to quantify the extent of inflammation at the appendiceal base, making it challenging to determine when applying a polymeric clip would pose difficulty. Future studies should focus on preoperative CT findings of inflammation in the appendiceal base, for which polymeric clips are difficult to apply. Third, although there was no statistical difference in surgical fee, the actual price of two pieces of the polymeric clips is cheaper (Gems-clip Plus, ₩7,100 each) than that of one endoloop (Gemsloop-PGLA, ₩33,260). However, there are usually six pieces of polymeric clips (GEMS clips) in a bundle. Therefore, the price for one operation is ₩42,600. We certainly have to purchase a reusable clip applier (₩243,000), but if there is a smaller bundle of fewer than six pieces, the price competitiveness can be better.

## Conclusion

The use of a polymeric clip is easier and more comfortable than the use of an endoloop in patients with uncomplicated appendicitis in the absence of appendiceal base inflammation. Even if the application of the polymeric clip fails, it can be compensated for using an endoloop or endostapler. Therefore, we suggest considering using the polymeric clip first if the base thickness on preoperative CT is less than 12 mm.

## Abbreviations

CT: Computed tomography
DRG: Diagnosis-related group payment
RCT: Randomized controlled trial
IRB: Institutional review board
IQR: Interquartile range
